# Candidate-gene based GWAS identifies reproducible DNA markers for metabolic pyrethroid resistance from standing genetic variation in East African *Anopheles gambiae*

**DOI:** 10.1038/s41598-018-21265-5

**Published:** 2018-02-13

**Authors:** David Weetman, Craig S. Wilding, Daniel E. Neafsey, Pie Müller, Eric Ochomo, Alison T. Isaacs, Keith Steen, Emily J. Rippon, John C. Morgan, Henry D. Mawejje, Daniel J. Rigden, Loyce M. Okedi, Martin J. Donnelly

**Affiliations:** 1 Department of Vector Biology, Liverpool School of Tropical Medicine, Pembroke Place, Liverpool UK; 2 School of Natural Sciences and Psychology, Liverpool John Moores University, Liverpool, UK; 3 Genome Sequencing and Analysis Program, Broad Institute of MIT and Harvard, Cambridge, USA; 4 Epidemiology and Public Health Department, Swiss Tropical and Public Health Institute, Basel, Switzerland; 5 University of Basel, Basel, Switzerland; 6 School of Public Health and Community Development, Maseno University, Maseno, Kenya; 7 KEMRI/CDC Research and Public Health Collaboration, Kisumu, Kenya; 8 Infectious Diseases Research Collaboration, Kampala, Uganda; 9 Institute of Integrative Biology, University of Liverpool, Liverpool, UK; 10 National Livestock Resources Research Institute, Tororo, Uganda; 11 Malaria Programme, Wellcome Trust Sanger Institute, Hinxton, Cambridge UK

## Abstract

Metabolic resistance to pyrethroid insecticides is widespread in *Anopheles* mosquitoes and is a major threat to malaria control. DNA markers would aid predictive monitoring of resistance, but few mutations have been discovered outside of insecticide-targeted genes. Isofemale family pools from a wild Ugandan *Anopheles gambiae* population, from an area where operational pyrethroid failure is suspected, were genotyped using a candidate-gene enriched SNP array. Resistance-associated SNPs were detected in three genes from detoxification superfamilies, in addition to the insecticide target site (the Voltage Gated Sodium Channel gene, *Vgsc*). The putative associations were confirmed for two of the marker SNPs, in the P450 *Cyp4j5* and the esterase *Coeae1d* by reproducible association with pyrethroid resistance in multiple field collections from Uganda and Kenya, and together with the *Vgsc*-1014S (*kdr*) mutation these SNPs explained around 20% of variation in resistance. Moreover, the >20 Mb 2La inversion also showed evidence of association with resistance as did environmental humidity. Sequencing of *Cyp4j5* and *Coeae1d* detected no resistance-linked loss of diversity, suggesting selection from standing variation. Our study provides novel, regionally-validated DNA assays for resistance to the most important insecticide class, and establishes both 2La karyotype variation and humidity as common factors impacting the resistance phenotype.

## Introduction

Insecticide resistance is a strongly selected, highly heritable trait that represents an excellent model system for the study of contemporary evolution^1^. Disease-transmitting mosquitoes are often subject to intense insecticidal selection pressure and in many instances there are critical financial and health implications of insecticide resistance evolution. In sub-Saharan Africa, where over 90% of all malaria fatalities occur, cases have fallen dramatically, due largely to the massive scale-up of insecticidal interventions such as treated bednets and indoor residual spraying^2^. Few insecticides are licenced for vector control and extensive application in public health, agriculture and household pesticide formulations has selected for widespread resistance to pyrethroids, by far the most important class of insecticides which may be used on bednets^3^. Whilst epidemiological evidence for an impact of resistance on malaria remains sparse^4^ there exists the worrying spectre that the recent gains in malaria control may be lost^5^. Preservation of pyrethroid efficacy is thus a primary objective for malaria control programmes, which need to apply effective insecticide resistance management. Insecticide resistance management depends on timely information on changes in resistance and the potential for cross resistance between insecticide options. High throughput molecular diagnostics can provide more sensitive, rapid and geographically-widespread assessments of changes in resistance than phenotypic assessments^6^. Knowledge of the genetic mechanisms underlying metabolic cross-resistance, which is far more difficult to predict a priori than cross-resistance at shared target sites^7^, is vital to allow implementation of insecticide combinations which will slow resistance development or allow susceptibility to return. Thus, improved knowledge of pyrethroid resistance mechanisms and provision of DNA markers for rapid predictive diagnosis^8^ and their evolution is an important goal to aid future insecticide deployment strategies.

Insecticide resistance occurs through four general mechanisms: insecticide target site alterations; elevated insecticide metabolism/sequestration; which are the primary focus here, and, potentially important but currently less-well described, reduced cuticular penetration and behavioural avoidance^9^. Target site resistance typically occurs through amino acid-altering substitutions, with the same mutations often found across diverse insect taxa^10,11^, or through copy number variation of alleles harbouring resistance mutations^12,13^. In *Anopheles* mosquitoes, and insects generally, pyrethroids and the organochlorine DDT target the voltage-gated sodium channel (*Vgsc*), within which knockdown resistance (*kdr*) mutations occur that can interfere with insecticide binding, preventing the normal insecticidal effect of repetitive nerve firing, paralysis and death^14,15^. In the primary malaria mosquito *Anopheles gambiae kdr* mutations exhibit limited variation in flanking haplotypes^1,16,17^. This conforms to a model of insecticide resistance evolution via hard selective sweeps from rare origins^18^, further supported by observed rapid increases of *kdr* variants toward fixation over a few years^16,19,20^. Similarly pronounced genomic footprints at the other major insecticide target site genes, acetylcholinesterase, AChE^13^ and GABA^21^ suggest that hard sweeps may be the norm for major resistance mutations in these essential neurotransmission genes. Such genomic signatures can greatly aid detection of major target site variants in genome scans, though selection toward fixation progressively reduces minor allele frequency and, as a direct consequence, statistical power to detect phenotypic association^6,22^.

With evidence of strong selection of target site mutations, it is perhaps surprising that metabolic resistance is usually considered the greater threat to vector control^23^. Indeed, the only widely accepted case of pyrethroid failure in malaria control to date was attributed to a local resurgence of *An. funestus*^24,25^ resistant to insecticide as a result of elevated expression of cytochrome P450 enzymes that metabolize pyrethroids^26,27^. Transcriptomic experiments conducted in *Anopheles spp*., have repeatedly linked overexpression of *CYP6* subfamily P450 genes to insecticide resistance and shown that some genes have the ability to metabolize pyrethroids or even multiple insecticides^28–30^. In contrast, there are few studies in *Anopheles* which have identified DNA variation associated with metabolic resistance. The exceptions have targeted a single candidate gene for DDT resistance^31,32^ or a pair of duplicated P450 genes^33^. A paucity of resistance-associated DNA variants might seem to indicate a relatively greater role for gene overexpression in metabolic insecticide resistance evolution. Yet no regulatory variants for insecticide resistance-linked differential expression have been identified in *Anopheles* populations. This implies that allelic variants involved in metabolic resistance, whether altering proteins or regulatory regions, remain to be discovered.

Significant methodological challenges are involved in the identification of metabolic SNPs, such as phenotyping and genotyping sufficient mosquitoes to attain statistical power; population substructure within samples^22^ and typically extremely short linkage disequilibrium in the *An. gambiae* genome^34^, which reduces correlations between functional and marker polymorphisms^35^. An additional problem for identification of markers for metabolic resistance may result from their flexibility of function and exceptionally high polymorphism, at least in the largest detoxification gene superfamilies^36^. Both of these properties contrast markedly with highly-conserved target site genes subject to purifying selection^13^, and suggest that selection at detoxification genes is more likely to act upon standing genetic variation and result in soft selective sweeps, making their detection far more problematic^18^.

The aim of our study was to apply a candidate gene-enriched single nucleotide polymorphism (SNP) genotyping array to identify putatively pyrethroid resistance associated polymorphisms segregating within a wild population of *An. gambiae* from the Uganda-Kenya border area, an important focus of East African pyrethroid resistance^37–39^, with association established by reproducibility in additional field populations.

The study proceeded via the following steps:

1. Collection of wild blood-fed females and rearing of progeny as isofemale lines.
2. Genotyping of adult females individually from the collections using the SNP array to investigate sources of within population structure
3. Bioassays and genotyping (using the same SNP array) of the each isofemale lines as a pools and association analysis based on relationship between family resistance phenotype and SNP genotypes
4. Identification of putative candidates with additional analysis first within a laboratory colony established from the same location and subsequently in several distinct wild populations
5. Sequencing and functional modelling analyses to attempt to identify causal SNPs and interpret the nature of selection

Our results suggest an important role for metabolic gene variants in pyrethroid resistance, which may be partially diagnosed using the SNPs identified as resistance-associated, and also indicate association of environmental humidity with pyrethroid resistance.

## Results

### Resistance phenotype

Isofemale families of *An. gambiae* from Tororo, Uganda were bioassayed using WHO permethrin papers for 60 min for males (N = 76 families) and 75 min for females (N = 98 families), which provided a full spectrum of family mortality levels ranging from zero to 100%, with a median of 76% (Figure S1). Humidity and temperature varied during the testing period in the field insectary (Figure S2), and across all families, mortality exhibited a significant inverse correlation with humidity (r = −0.43, P = 4 × 10^−6^; Figure S3), which was subsequently treated as a covariate in association analysis. Within each family, male and female mortalities were strongly correlated (r = 0.65; P = 2 × 10^−9^), and remained so following correction for humidity (partial r = 0.61; P = 3 × 10^−6^), suggesting a strong familial basis to resistance. Females were used for subsequent DNA analysis taking the proportionate mortality of the family from the bioassays (Figure S1) as a quantitative resistance phenotype.

### Within population structure

**Figure 1:**
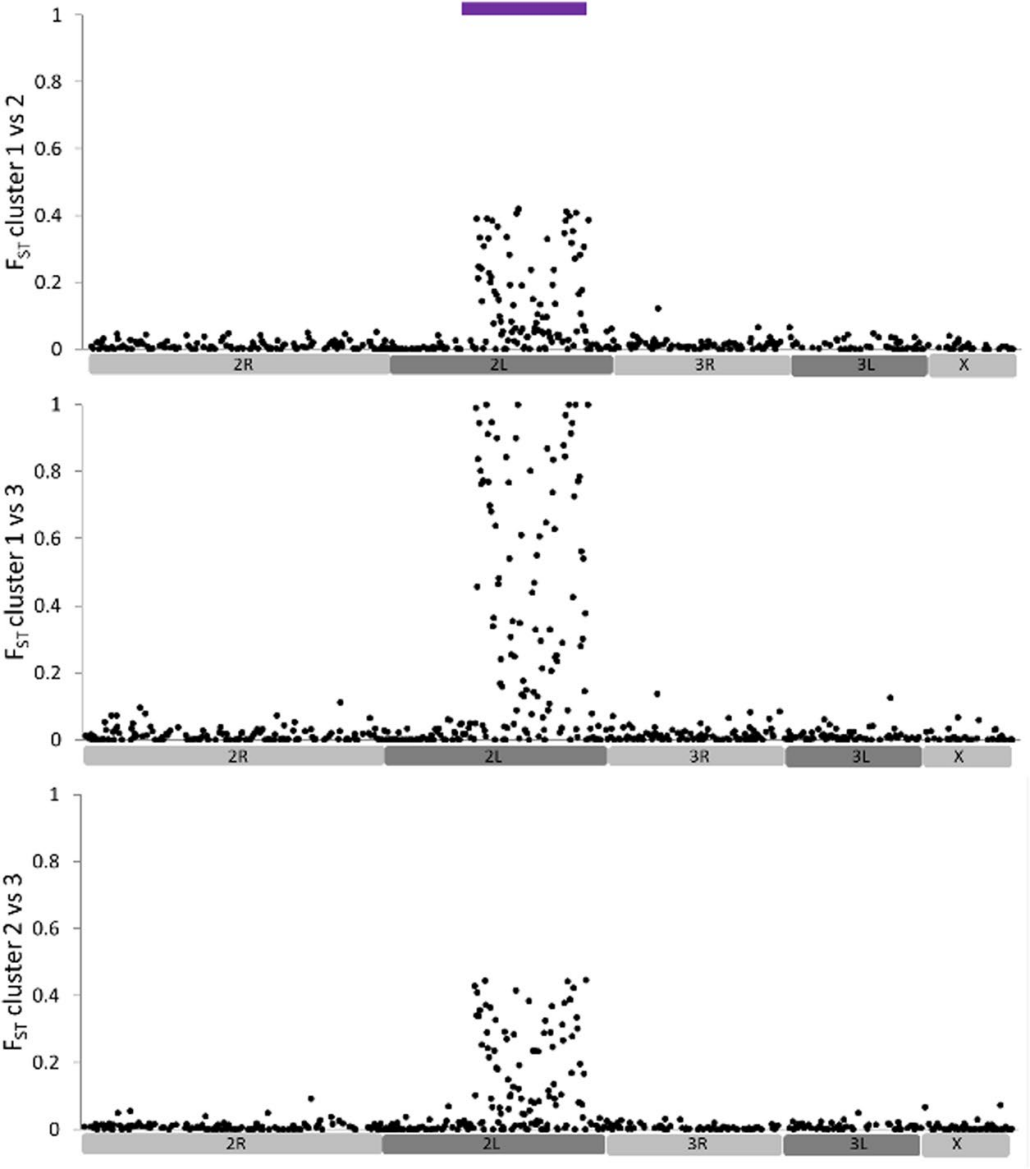
Analysis of sources of within-population structure in individually-genotyped adult females. Plots show F_ST_ values for each SNP compared among each of the three clusters identified using BAPS. The upper bar shows the location of the 2La inversion region.

We first examined evidence for within-population structure using individual genotypes from adult female *An. gambiae*, which comprised a collection of the wild caught mothers of the isofemale lines and others which did not produce sufficient offspring for use. These females were not classified by phenotype and were used solely for assessment of possible sources of within population structure and relatedness, which might impact subsequent association analysis using the pooled isofemale lines. Clustering analysis suggested three partitions, differentiation between which showed a strong localisation to the large 2La inversion region, with very limited inter-cluster divergence elsewhere in the genome (Fig. 1). Levels of differentiation within the 2La region were consistent with comparison among two homokaryotypic clusters and a heterokaryotypic cluster, and linkage disequilibrium was also extreme (Figure S4). This suggests that 2La inversion polymorphism was the only major source of structure within the sampled population, and that the sample did not comprise of groups of closely-related individuals. Though clear that 2La polymorphism is a major source of genomically-localised subdivision, it was unclear how it might influence association analyses because these females were not phenotyped. Therefore this was evaluated using principal components analysis as part of the association analysis of isofemale lines.

### Association analysis using isofemale lines

For pooled genotyping data from the isofemale lines, estimated allele frequencies were highly correlated with those calculated from individual genotypes after correction for SNP-specific dye bias (R^2^ = 0.98; Figure S5). Principal components analysis was used to identify covariates indicative of stratification in the pooled data based on 195 control (non-candidate gene) SNPs. The first principal component (PC1) was significantly correlated with resistance phenotype (partial correlation controlling for humidity: r = −0.33, P = 0.001; Table S1). PC1 primarily reflected variation within the 2La inversion region. All control (non-candidate) SNPs correlating strongly with PC1 (r≥0.5, N = 13) were located therein, and the mean correlation for 2La SNPs (r = 0.41, N = 32, was significantly higher (Mann-Whitney U-test, Z = 5.30, P < 0.001) than for control SNPs distributed throughout the rest of the genome (r = 0.11, N = 163). A second principal component was also correlated with mortality (r = 0.22, P = 0.03; Table S1), but showed no localisation of SNPs within the genome, and no difference in correlation between SNPs in 2La and elsewhere (Z = 1.45, P = 0.15). Owing to the concordance with individual genotyping results (above), in which the 2La region appeared to be the major source of stratification within the pooled dataset, we opted to consider PC1 further in order to apply correction for 2La variation. Elevated probabilities within the 2La region were evident when plotting all SNPs, but were removed by correction for PC1 as a covariate (Figure S6A), whereas if SNPs from the 2La region were removed *a priori*, correction for PC1 was not required to align the vast majority of SNPs with their expectation (Figure S6B). However, because correction for 2La variation (via PC1) might obscure SNPs which contributed to, or acted in addition to, the resistance association of 2La, and also because correction is likely to be imperfect, we considered results both with and without correction for PC1.

**Figure 2:**
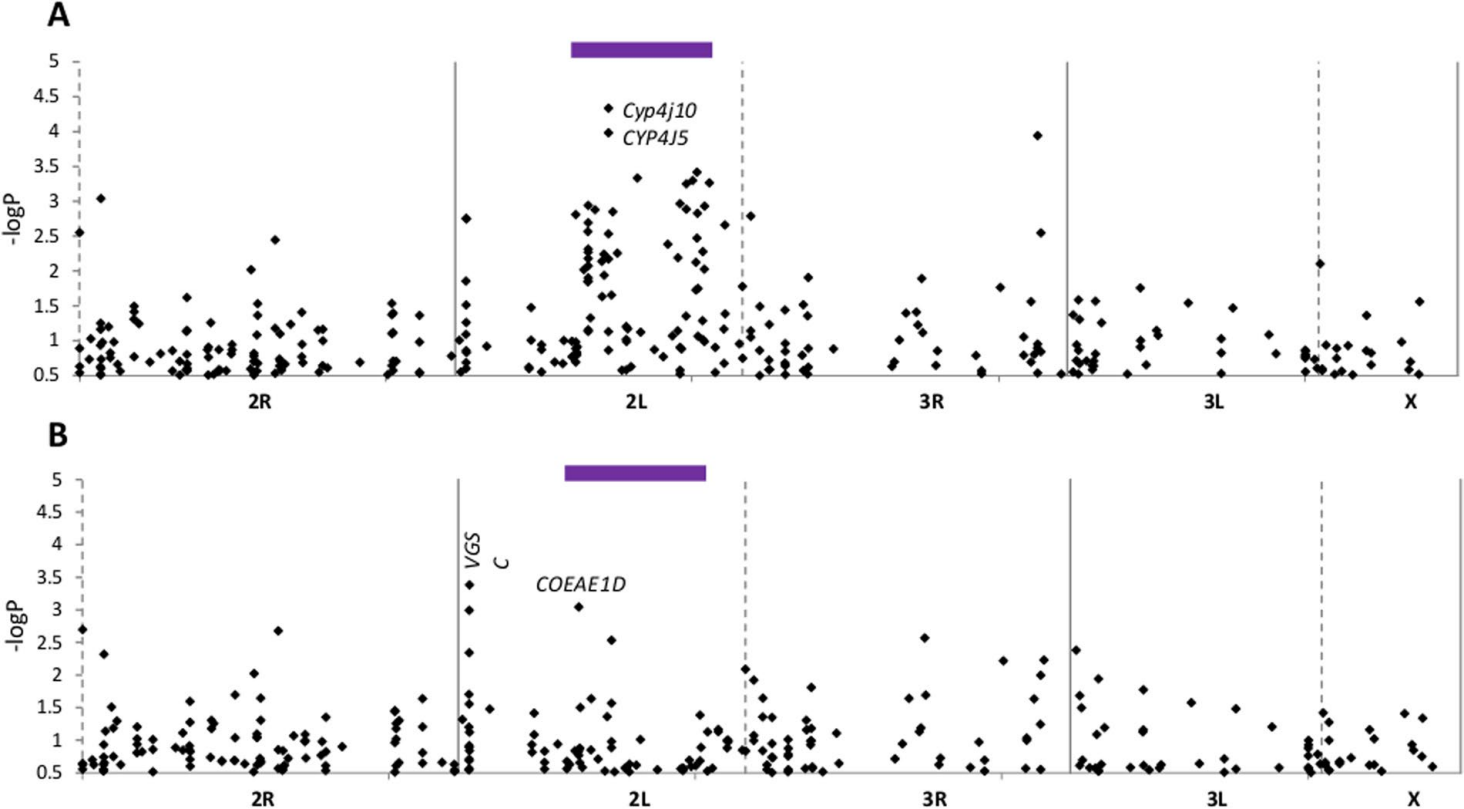
Family pool association analysis for permethrin resistance (**A**) corrected for humidity as a covariate; (**B**) corrected for humidity and PC1 (a proxy for 2La polymorphism; see Fig. 1). Test probabilities are shown for each SNP arranged on a physical scale across chromosomes, centromere positions are shown by solid vertical lines; chromosome breaks by dashed lines. The purple bar indicates the 2La inversion region.

For the association analysis the relatively limited number of families analysed limited study power, with calculations suggested only moderate-large effects might be detected (Materials and Methods). Consequently, we did not apply formal thresholds for significance in the analysis, but rather identified candidate SNPs for further investigation from those with high ranking -logP values, with demonstration of association based on subsequent replication of results. Most of the SNPs with lowest P-values were on chromosome 2L, of which the two showing strongest evidence for association were a synonymous variant in *Cyp4j5* and a non-synonymous variant in *Cyp4j10* located 2kb apart (Fig. 2A; Table S2). Systematic elevation of P-values was clear within the 2La inversion, especially toward the ends, and following correction for PC1, P-values of most SNPs located within 2La reduced substantially. SNPs with the lowest P-values post-correction were from chromosome 2L but outside the 2La region; within the *Vgsc* target site gene and a carboxylesterase gene, *Coeae1d* (Fig. 2B).

The candidate P450 and carboxylesterase gene SNPs were at high minor allele frequency (MAF, 0.35–0.49). However, those in the *Vgsc* exhibited much lower MAF (≤0.12), possibly as a result of a selective sweep of the *kdr 1014S* mutation, which is close to fixation (frequency = 0.94). To address the issue of potentially poor detectability of association of low MAF SNPs that might be influenced by strong selection in the wild, we performed an additional (individual-mosquito) association study using the class II pyrethroid deltamethrin. This experiment used bioassay survivors and dead from a recently-founded colony from the same location, which had not been previously been exposed to insecticide (Figure S7A). In the colony, MAFs of *Vgsc-*L1014S (MAF = 0.37) and other *Vgsc* SNPs were much higher than in the wild population (Table S3) and L1014S exhibited the strongest resistance association of all the SNPs typed (Figure S7B). The *Vgsc* SNP exhibiting the lowest probability in the previous analysis (Fig. 2B) and the SNP in *Coeae1d* were also strongly associated with resistance phenotype in the colony.

### Marker association repeatability

**Figure 3:**
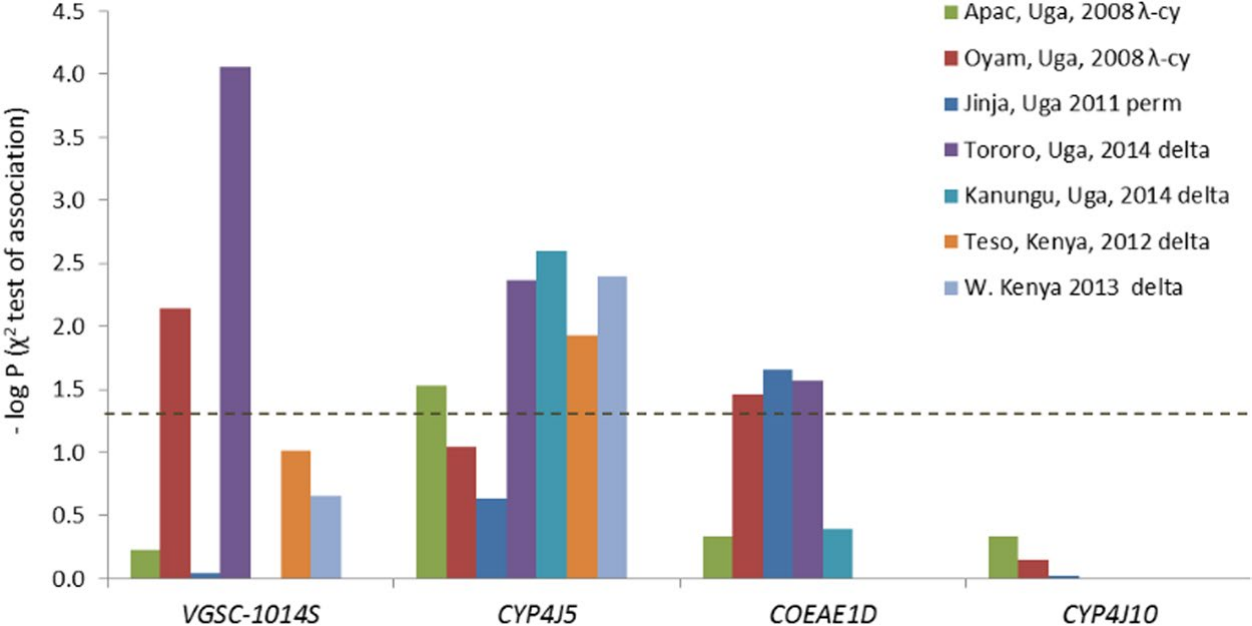
Repeatability of SNP associations in independent field samples from Uganda and Kenya with three different pyrethroid insecticides (see Table S4 for full results). The dashed line indicates P = 0.05. Uga = Uganda; insecticides used for bioassays: λ-cy = lambda cyhalothrin; delta = deltamethrin; perm = permethrin. The year of collection is shown.

We chose four SNPs for further testing, the linked *Cyp4j5* and *Cyp4J10* variants, the *Coeae1d* SNP and the *V*gsc-L1014S variant. When tested sequentially using stepwise regression, insectary humidity explained the highest proportion of the variance in permethrin resistance phenotype among the families, but each of the four SNPs explained significant additional variance, together totalling 22% among isofemale lines, with *Cyp4j5* the strongest predictor (Table 1). Novel TaqMan assays were designed for the *Cyp4j10*, *Coeae1d* and *Cyp4j5* SNPs. For the latter we used a non-synonymous SNP (not present on the array) in highest LD with the original marker, a leucine-phenylalanine change at codon 43 of the gene (L43F) (see Materials and Methods for details of source). These assays were combined with that for *Vgsc-*L1014F for screening in up to seven independent field samples phenotyped for susceptibility to three pyrethroid insecticides (Fig. 3). The known causal marker *Vgsc*-L1014S was significantly resistance-associated in two of the samples and the *Coeae1d* marker in three. The *Cyp4j10* SNP failed to approach significance in three test samples and screening was discontinued, but the *Cyp4j5-*L43F marker was highly reproducible, with significant resistance association in five out of seven independent sample sets from both Uganda and Kenya (Fig. 3). Average effect sizes and their variability are shown in Table 2 and Table S4. For *Vgsc*-L1014S, odds ratios were highly variable, ranging from approximately 1 to over 10; were more consistent for *Cyp4j5* (range 1.5–4.7) and lower but again quite consistent (range 0.7–2.7) for *Coeae1d* (Table S4). For *Cyp4j5* we also investigated the predictive utility for a more extreme phenotype by comparing deltamethrin survivors of a 2 hour exposure with those killed by a one hour exposure; interestingly this yielded the highest odds ratio observed for this marker (OR = 6.9, P = 0.0001, Table S4).

**Table 1:** Stepwise regression of pyrethroid resistance by the environmental correlate humidity and candidate SNPs among families of the Tororo discovery population.

chromosome	gene type	minor allele frequency	predictor	model r^2^	P-value
	environmental correlate		1. Humidity	0.167	0.00002
2L	metabolic (P450)	0.37	2. *Cyp4j5*	0.284	0.00009
2L	target site (*Vgsc*)	0.06	3. L1014S	0.335	0.005
2L	metabolic (COE)	0.49	4. *Coeae1d*	0.363	0.026
2L	metabolic (P450)	0.45	5. *Cyp4j10*	0.393	0.021

The number in the predictor column indicates the order of entry of the SNP into the model.

**Table 2:** Allele frequencies and association with resistance measured by odds ratios for the four candidate SNPs in populations from Kenya and Uganda in which reproducibility was tested.

SNP in gene	Populations genotyped	Total allele count	Mean resistant allele frequency (st. dev)	Mean odds ratio (st. dev)
*Cyp4j5*	7	1370	0.61 (0.13)	2.94 (1.10)
*Vgsc*	7	1068	0.84 (0.22)	3.59 (3.71)
Coeae1d	5	738	0.53 (0.04)	1.93 (0.87)
Cyp4j10	3	502	0.47 (0.05)	1.04 (0.24)

Standard deviations are measured across populations.

Temporal analysis of variation in marker frequencies in Tororo in 2013–2014 revealed relative stability of frequencies of each of the three metabolic gene markers (Figure S8). Interestingly, genotype frequencies of the *Cyp4j5* and *Cyp4j10* markers were strongly correlated (Table S5) as expected from their very close proximity in the genome, suggesting that the initial association of the *Cyp4j10* marker for pyrethroid resistance may have in part arisen from co-variation with *Cyp4j5*. Neither *Cyp4j5* nor *Coeae1d* marker genotype variation was correlated with 2La variation, despite apparent allele frequency covariation (Figure S5) highlighting their independent predictive utility (Table S5).

### Investigation of possible causal SNPs

The resistance-associated variant at base 20288132 (VectorBase nucleotide numbering) in the *Coeae1d* gene is a synonymous variant. Therefore, to investigate whether any non-synonymous SNPs showed evidence of association which might be underpinning this, we sequenced a single individual from each of 24 families from the extremes of the mortality distribution (see Figure S1A; Table S6A). The *Coeae1d* gene is extremely polymorphic with a large number of missense variants (N = 116), many of which are at high frequency (31% with MAF > 0.1; 19% with MAF > 0.2). In this methodologically-independent replication of the initial association analysis, the 20288132 marker SNP exhibited a significant level of association (OR = 2.9, χ^2^ =  5.20, P = 0.022) but this was not exceeded by any function-altering polymorphism within the gene, and only marginally so by two other synonymous SNPs (Figure S9; Table S6B). We also genotyped the 2La inversion in the same samples, which showed significant association of the derived (2L+^a^) karyotype with permethrin resistance (OR = 4.0, χ^2^ =  4.85, P = 0.028).

**Figure 4:**
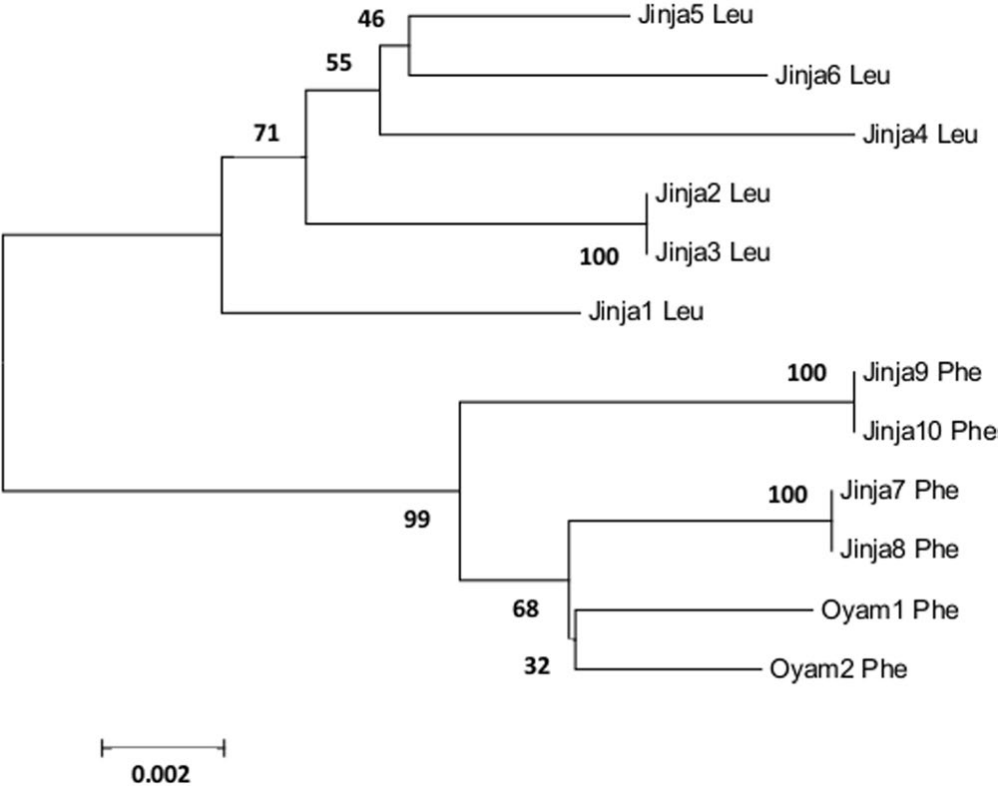
Evolutionary relationships of the resistant (Phenylalanine) and susceptible (Leucine) alleles of *CYP4J5* marker L43F in Ugandan samples. The optimal tree with sum of branch lengths = 0.062 is shown. Percentage bootstrap values (1000 replicates) are shown next to branches. The sample prefix indicates mosquito origin (Jinja or Oyam, Uganda).

Sequencing of the *Cyp4j5* gene in Ugandan individuals revealed a split between resistant and susceptible haplotypes around the L43F variant, including similarity of resistant alleles from distinct locations (Jinja and Oyam) in southern and northern Uganda (Fig. 4). However, no marked difference in diversity, as would be expected with a strong selective sweep, was evident. We used structural modelling to evaluate the possible functional consequences of the L43F, and other non-synonymous substitutions in *CYP4J5*. Of the variants (Table S7) that could be modelled (noting that available templates bore only approximately 20% sequence identity to CYP4J5, limiting model quality), the substitution at L43F was not modelled reliably but seems unlikely to have an impact on protein stability. Of the other SNPs only one, a rare Alanine to Threonine substitution at codon 69, was predicted to potentially fall near enough to the active site for impact. However, this SNP was not detected as being in LD with the original marker, nor with the L43F variant; in fact the correlation with each was negative suggesting, if anything, weak linkage of the resistance associated allele at each marker with the wild type allele at A69T. Strong repeatability of the L43F mutation as a resistance predictor (above) suggests that either this is in strong LD with an, as yet unidentified, causal variant, or contributes directly to the resistance phenotype in a way that was not revealed by the current modelling approach.

## Discussion

Using a candidate-GWAS approach we have identified two novel SNPs in genes from major metabolic superfamilies that are reliably and reproducibly associated with resistance to class I and II pyrethoids. We also detected a strong relationship between bioassay survival and humidity in our initial experiment and evidence for association of 2La inversion polymorphism with resistance. Moreover, although the genes containing the associated SNPs are located within and close to 2La, respectively, independent temporal dynamics of the SNP and 2La polymorphisms in Ugandan field samples suggests that additional resistance-associated variants remain to be discovered within 2La. The SNP markers identified are unlikely to be causal substitutions and we speculate that linkage disequilibrium between each marker SNP and one or more haplotypes, each likely to contain multiple non-synonymous changes in a resistance-conferring haplotype, may underpin the associations observed. Certainly the pattern of polymorphism deviates from that arising from selection on a single, relatively recent mutation^13,16,17^ and, particularly if haplotypes are driving the resistance association, selection from standing variation appears more likely^40^.

In the Eastern Ugandan Tororo field population strong association of the *Vgsc*-1014S (*kdr*) variant with both permethrin and DDT resistance has been demonstrated previously^37^ and we therefore suspected that the relatively weak association with permethrin-survival across families in our study might be attributed to limited statistical power arising from low polymorphism (1014S frequency ≈ 95%). Results from the Tororo colony, within which the frequency of the wild type 1014L allele was much higher, supported this hypothesis: L1014S was the SNP most strongly associated with survival following deltamethrin exposure. This is significant because: (i) the association of *Vgsc-*1014S has not previously been tested against a background of many other candidates; (ii) a significant link with class II resistance has proved difficult to establish^37,41^; (iii) it suggests that 1014S, and probably other *kdr* mutations continue to play a pivotal role in resistance in the presence of other resistance-linked mutations. Thus, it is important to recognise that even following rise toward fixation where *kdr* mutations explain relatively little of the variation in resistance *within* a population, they provide a strong baseline of resistance, which can be elevated further – rather than being superseded - by the presence of other resistance-linked variants^6^. An additional *Vgsc* mutation (N1575Y) detected in *A. gambie* and *A. coluzzii* across West Africa^17^ illustrates this point. The 1575Y allele occurs only on a *Vgsc*-1014F background and though the SNP itself exhibits a moderate odds ratio (≈2), this effectively doubles that of *kdr1014F*, and so translates into a large aggregate odds ratio (≈12)^7,17^.

Polymorphism of the 2La inversion has long been linked with important phenotypic variation in *An. gambiae*, primarily with adaptation to environmental aridity^42,43^, but also with refractoriness to *Plasmodium falciparum*^44^. Our data suggest that insecticide resistance can also be associated with the 2La inversion, with the major permethrin resistance-associated principal component (PC1) identified in our family pool study, correlating strongly with variation at SNPs within 2La. Owing to the known relationship between the 2La inversion and aridity, it is important to note that in the Tororo discovery dataset the association of PC1 remained significant following correction for humidity, i.e. both humidity and 2La polymorphism were determinants of resistance phenotype. In addition, when 2La polymorphism was typed directly, during our investigation of *Coeae1d* variation, the 2L+^a^ karyotype was significantly associated with permethrin survival. Genomic stratification in the Tororo population was attributable to 2La polymorphism, therefore we opted to investigate further the most associated SNPs that emerged prior to, and post-correction using PC1 as a proxy for 2La variation. The resultant analyses showed that identification of top candidate SNPs solely post-correction for 2La would have been a mistake, because the strongest and most reproducible candidate SNP (*Cyp4j5*-L43F) would probably have been missed at the discovery phase. This is noteworthy and reflects a difference in genomewide stratification as expected in an admixed population, which would necessitate *a priori* correction to avoid false positives, and genomically-localised stratification, as seen here, which is a driver of the phenotype and unrelated to mating barriers.

Interestingly, when typed in a series of field samples collected from near Tororo between 2013 through 2014, neither *Cyp4j5* (which was correlated with PC1) nor *Coeae1d* (uncorrelated with PC1) genotype frequencies were correlated within 2La karyotype variation, despite visual resemblance of summary data (see Figure S4). This is an important finding because it means that these two SNPs and 2La can serve as independent diagnostic markers, and perhaps most crucially because evolutionary dynamics of *Coeae1d* and especially *Cyp4j5* (located within 2La) need not be constrained by dependence on 2La inversion polymorphism dynamics, which might be subject to multiple and potentially contrasting selection pressures. Whether such constraints are currently operative within the East African area we covered in our study is unclear, but recent indirect evidence suggests that insecticidal pressure might be the dominant selective factor for 2La variation. In *A. gambiae* s.s. samples from a 17-year time series collected in Western Kenya, Matoke-Muhia *et al*.^45^ detected a sharp decline in frequency of the previously numerically-dominant 2La karyotype in favour of the 2L+^a^ karyotype, which correlated strongly (r = −0.96) with the changing coverage of pyrethroid-treated bednets in the area. To our knowledge our study is the first to provide direct association data directly linking pyrethroid resistance with 2La inversion polymorphism, adding corroborative evidence to the field survey results from Western Kenya.

An important objective is now to identify the genes/variants in 2La underpinning pyrethroid resistance association (noting that *Cyp4j5* apparently is not the major cause). Contrasting with most of the *An. gambiae* genome^22,34,46^, LD within the 2La inversion region can be extreme, especially for a few megabases within the breakpoints around 22 and 42Mb^22^. Such strong LD creates the problem, commonly encountered in GWAS of human populations outside of Africa^35^, of how to isolate markers from large statistically-elevated haplotype blocks. Increasing sample size may be one answer, but will prove difficult in the near-term, because low LD throughout the majority of the genome argues strongly for the use of high density genotyping-by-sequencing approaches for which highly powered studies remain expensive. Instead, we suggest that investment of greater resources into *post hoc* investigation of repeatability of phenotype associations for smaller, filtered, sets of markers in wild populations could permit the signals from multiple linked markers to be teased apart. Polymorphic inversions are common in insects and low genomic LD in wild populations likely to be also, therefore such study design issues are of relevance to many taxa.

Our study demonstrates that pyrethroid resistance in eastern Uganda/western Kenya populations has a multivariate basis. In addition to the genetic polymorphism associations in the original discovery phase of the project, our data show a strong impact of humidity during bioassays on resistance, as reported previously in houseflies *Musca domestica*^47^. Although humidity varied naturally, rather than experimentally, in our study it explained over 17% of the variation among families in resistance and is very likely to be an important ecological determinant of insecticide resistance phenotypes under natural conditions. Of the genetic component of variation, both target site and metabolic variants play a role, with the two metabolic markers identified explaining a significant proportion (19%) of within-population variance. In a microarray study of the same Tororo population sampled the following year, relatively few significantly overexpressed genes were detected and only two detoxification genes^48^, though the design did not include a fully susceptible sample which will tend to accentuate discovery of overexpressed genes^7^. Nevertheless, available evidence suggests that the variants we identified are likely to link with mutations causing qualitative rather than quantitative protein variation, and we suggest that in Tororo this may be a more important determinant of pyrethroid resistant phenotypes. Owing to the paucity of agnostic DNA-based studies, which contrasts with the plethora of insecticide resistance-targeted gene expression experiments, whether this is population-specific, or the case more generally, remains to be determined.

## Conclusion

With increased selective pressure arising from massive scale-up of long lasting insecticidal net (LLIN) distributions over the last decade, levels of pyrethroid resistance are likely to increase further and threat to control appears imminent. Identification of the genetic mechanisms leading to heightened resistance, and provision of simple assays for rapid diagnosis will aid attempts to manage resistance and retain pyrethroids as a viable option. Our study has highlighted the importance of allelic variants, outside the insecticide target site, as determinants of a significant fraction of variation in the resistance phenotype in a wild *An. gambiae* populations. Whole genome resequencing should permit identification of a spectrum of DNA polymorphisms underpinning resistance, and facilitate development and widespread application of DNA diagnostics as an accurate proxy for *a priori* assessment of phenotypic resistance.

## Materials and Methods

### Sample collections

Indoor-resting adult females were collected by aspiration from houses in Tororo, Uganda (00°40′41.6″N, 34°11′11.6″E) in November 2008 and transported to insectaries at the nearby National Livestock Resources Research Institute (NaLiRRI) facility. Morphological examination of almost 900 adult females revealed that the vast majority of samples (≈98%) were *Anopheles gambiae s.l*., with the remainder *An. funestus*, which were discarded. Adult female *An. gambiae s.l*. were held individually in plastic cups covered with fine netting, and contained moist cotton wool at the base covered by a disc of filter paper onto which eggs were laid. A cotton wool pad on top of each cup provided 10% sucrose solution *ad libitum*. Once eggs were laid, each filter paper disc was removed to a plastic bowl containing water, the adult female was sacrificed and preserved over silica gel. Following hatching, larvae were fed twice-daily with finely-ground Tetramin fish food. Pupae were transferred to netted cups containing a small volume of water, with one cup per isofemale family. On eclosion females and males were separated daily and isofemale line-specific bioassays were performed separately on 10–20 (median N = 15) 3–5 day old adult females (or males) in WHO tubes using standard 0.75% permethrin papers. Informed by bioassay data from preliminary tests on mixed F_1_ offspring from multiple families, we used an insecticide exposure time of 75 min (and 60 min for males), which aimed to produce an intermediate level of mortality (ca. 50%) for the population as a whole. Humidity and temperature were recorded during each assay. Approximately 24 h after exposure, all individuals were counted as alive or dead and preserved individually over silica gel.

For establishment and phenotype-classification of a colony for secondary association analysis, eggs were initially transported to the Liverpool School of Tropical Medicine from the Tororo collection site in December 2008; with a second supplementary collection added in May 2009. The colony was maintained without any insecticide exposure until September 2009 at which point 3–5 day old adult females were exposed to varying concentrations of the pyrethroid insecticide deltamethrin (Greyhound chemicals, UK) using WHO bioassay tubes and custom papers produced using the appropriate quantity of deltamethrin dissolved in acetone, added to silicone oil and spread over Whatman papers. Following the usual 24 h holding period, 20 adult females that died at low deltamethrin concentrations (<0.01%) and 36 surviving high deltamethrin concentrations (≥0.05%; note that 0.05% is the standard WHO resistance diagnostic concentration) were defined as susceptible and resistant classes, respectively and preserved individually over silica gel.

### SNP array genotyping

DNA from *An. gambiae s.l*. individuals was extracted, quantified and typed to species using previously-described methods^49^. More than 96% of samples were *An. gambiae* (formerly *An. gambiae s.s*. S-form); the remainder were *An. arabiensis*, which were not considered further. The Illumina Goldengate 1536-SNP array used is described in detail elsewhere^22,49^ but briefly was enriched for SNPs at 266 candidate detoxification or target site genes, with approximately 20% of SNPs, located in intergenic regions or non-candidate genes. Although the SNP-array was populated by candidate genes, it should be noted that none of the SNPs present were known candidates in this geographical area. The genotype of the known resistance mutation, *Vgsc* (*kdr*) *L1014S* was also determined by direct sequencing of PCR products amplified using primers ex20+ F/R^16^. Mothers of isofemale lines and additional females collected but not producing eggs were genotyped individually, as were females from the laboratory colony. Female offspring from families were genotyped as single-family pools, using an equimolar amount of DNA from each individual to provide a final concentration per family pool of 50 ng/µl.

### SNP array data analysis

#### Individuals

Genotyping arrays for 216 adult females (N = 98 mothers of the isofemale lines and N = 118 other females from house collections) and N = 56 Tororo colony specimens were scored using Beadstudio v3.2 (Illumina Inc). Haploview 4.1^50^ was used to compute minor allele frequencies, linkage disequilibrium among SNPs as r^2^, observed and expected heterozygosities, and to test for deviation from Hardy-Weinberg expectations. Individual cluster analysis of genotypes at the control (intergenic and non-candidate) SNP loci was performed by BAPS 5.2^51,52^, with multiple runs performed to obtain the optimum clustering solution.

#### Pools

Alleles labelled with different dyes seldom give a perfectly equal signal in heterozygotes and such dye bias may be assay-specific. Dye bias is typically not a major concern for individual genotyping but can greatly impact accurate estimation of allele frequencies from pooled DNA^53^. Therefore prior to analysis of pooled genotypes we computed the dye bias (*k*) for each SNP for which a minimum of two individual heterozygotes were available, as the mean cy3: cy5 dye ratio. For example, if heterozygotes exhibit a mean ratio of 0.5 (i.e. a twofold cy5 bias) then *k* = 0.5. We excluded SNPs exhibiting extreme or poorly estimated values of *k*, which can give rise to poor estimates of allele frequencies in pooled analysis^53^, based on two exclusion criteria: (1) *k* > upper 95^th^ percentile of the range of *k* values; (2) standard deviation of *k* across individuals > upper 95^th^ percentile of the range of *k* value standard deviations. Of the 1536 SNPs on the array 895 could be scored reliably, were polymorphic and exhibited *k* values meeting criteria 1 and 2: only these SNPs were used in the analyses. Raw X and Y (cy3 and cy5) signal data were extracted from Beadstudio v3.2 and used as the basis for all analyses of pooled DNA. Allele frequencies, p(X) and p(Y), were computed as:

1 $${\rm{p}}({\rm{X}})={\rm{raw}}\,{\rm{X}}\,({\rm{cy}}3){\rm{signal}}/[{\rm{raw}}\,{\rm{X}}({\rm{cy}}3){\rm{signal}}+k.{\rm{raw}}\,{\rm{Y}}({\rm{cy}}5){\rm{signal}}]$$

2 $${\rm{p}}({\rm{Y}})=1-{\rm{p}}({\rm{X}})$$

Principal components analysis (PCA) using allele frequency data from 195 control SNPs was used to investigate possible within-sample stratification using the pooled genotype data. Association analysis was performed on rank-transformed data by computing correlations between allele frequency and proportionate bioassay mortality for each family, or partial correlations to correct for possible covariates (temperature, humidity and principal components; see Results).

Power calculation suggested that our analysis could detect a slope for mortality vs allele frequency in the analysis of 0.46 (as significantly different from zero), with 80% power and a Bonferroni-corrected alpha level (0.05/N SNPs), with the 894 SNPs successfully genotyped and our dataset of pooled genotypes from 98 families. This suggests that moderate-large effect sizes associated with any individual SNP would be detectable. Whilst this was compatible with our aim to discover markers with useful predictive power for phenotypes (i.e. those of larger effect) rather than a comprehensive set of variants, owing to the limited power we do not apply formal significance thresholds in the analysis but rather base assessment of association on reproducibility of candidates selected for further testing.

IBM SPSS 22 was used to perform subsequent stepwise regression analyses for the pooled genotype data and multiple logistic regression for the individual data from the reproducibility testing populations.

### Associated gene sequencing

To attempt to identify the possible causal SNP and evaluate evidence of selection the *Cyp4j5* gene was sequenced in several individuals from Jinja and Oyam, distinct locations from southern and northern Uganda, respectively (locations given in subsection below). Specimens were genotyped using the *CYP4J5* marker (2L: 25635973) and six homozygotes for each allele *Cyp4j5*–43L and *Cyp4j5*-43F, the latter resistance associated, were selected for cloning and sequencing. The entire gene was amplified using primers 4J5F1 5′-AGCACCACGGTAAGGATGTC-3′ and 4J5R1 5′-GCGGAGAAACGTAACCCATA-3′ with a Phusion PCR kit (Thermo Scientific) prior to cloning in *E.coli* using a pJET1.2/blunt cloning vector. Due to the size of the product (~3.5 kb), two internal primers (4J5SEQ2 5′-ATTGCCGACTGTAGCTCGAT-3′ and CYP4J5-2 5′-GGCTTCTTTGGGACACACAT-3′) were used in addition to the pJET plasmid specific primers (pJETF and pJETR). Clones were sequenced by Macrogen, Korea and edited using CodonCode Aligner v4 (CodonCode Corporation). Gene trees were produced in MEGA 7^54^ using the neighbor-joining method with 1000 bootstraps. Evolutionary distances were computed using the Maximum Composite Likelihood method using all codon positions. All positions containing gaps and missing data were eliminated yielding a total of 1596 positions in the final dataset.

The *Coeae1d* gene was sequenced in a single individual from 24 families from each end of the spectrum of mortalities, and each individual thus designated as resistant or susceptible. Initial PCR amplification of a 2047bp fragment used primers Agap5756his (5′ATCGTCAACGTGCTCAGTCA3′) and Agap5756exp (5′AGCACAGCGACTAACTCTTGC3′) in the following mixture 5 μl KapaTaq 10X buffer, 1 μl (10mM dNTPs), 10 picomoles of each primer, 1.5 U KapaTaq polymerase, 40.7 μl water & 1 μl DNA template. Cycling conditions were 95 °C for 5 min, followed by 40 cycles of 95 °C for 30 sec, 57 °C for 30 sec, 72 °C for 90 sec, with a final step of 72 °C for 10 min. Products were cleaned with QIAquick PCR Purification Kit (Qiagen) and sent for sequencing by Macrogen, Korea using the following overlapping sequencing primers covering 1951 bp within the amplicon, which included the whole gene (5756SEQ7i 5′AACTCCTCGAAACCTCACCTA3′; 5756SEQ1 5′ATGCAACCCTTCCTAGCG3′; 5756SEQ2i 5′ACCCCATTTGCTCAAACA3′; 5756SEQ3i 5′GGTTTCGAGGAGTTTTGTTGT3′; 5756SEQ5 5′TCACTCCGGCAGCTTCCTA3′; 5756SEQ4i 5′CAACTCAACTCTCTTACAGACATGC3′). Sequences were aligned to a reference sequence from VectorBase in CodonCode Aligner v4, edited and polymorphisms identified. Chi-square tests were conducted for each SNP identified to investigate association with permethrin resistance phenotype, by comparing allele frequencies between resistant and susceptible individuals. We also genotyped polymorphism of the 2La inversion in these samples using a PCR-based method^55^ for association analysis based on chi-square tests as before.

### Functional modelling

Templates for modelling of *Anopheles gambiae* CYP4J5 were ranked by a search of the Protein Data Bank^56^ using HHsearch^57^. The best scoring 10 structures were used for comparative modelling using the recent high-resolution methods implemented in Rosetta^58^ to generate 30 models. The top model was used for interpreting the likely consequences of observed SNPs using structure-based methods FoldX^59,60^ and PoPMuSiC^61^. This analysis was confined to regions that comparison between models indicated were predicted reliably. SNPs positioned in regions not considered reliably modelled were interpreted using PolyPhen2^62^. The structure of deltamethrin, obtained from the Toxin and Toxin Target Database^63^, was manually positioned using PyMOL (www.pymol.org) in the CYP4J5 model in a similar position to ritonavir bound to CYP3A4^64^, one of the template structures. Its position relative to amino-acids altered by SNPs was assessed. Since the available templates bore only low (~20%) sequence identity with *Anopheles gambiae* CYP4J5 and apo-structures were among the templates, the binding cavity of the CYP4J5 model was not predicted accurately enough to allow for automated docking. Preliminary functional modelling of COEAE1D was performed but owing to a very large number of non-synonymous variants (see Results) was discontinued.

### Replication study samples and assays

We designed novel TaqMan assays for two candidate CYP450 markers identified in the family pooled genotyping and colony individual genotyping experiments and the *Coeae1d* marker. For the *Cyp4j5* marker, non-target polymorphism in the assay region prevented design for the associated SNP, therefore we sought to design an assay for a non-synonymous SNP within the gene in highest linkage disequilibrium (LD) with the marker. Data were obtained from whole genome sequences of 40 isofamily pools of females, collected in the same way as before from the same locations one year later^48^. LD was approximated by computing family-wise Pearson correlations for each of the variants identified with the original marker (Table S7). The SNP with the highest correlation coefficient (and similar minor allele frequency to the original marker) was a leucine to phenylalanine substitution at codon 43, for which an assay was successfully designed.

For independent replication of marker: phenotype associations, samples bioassayed for pyrethroid susceptibility were obtained from a number of spatially and/or temporally distinct collection locations in the East African region. Oyam (02°14′N 32°23′E), Apac (01°59′N 32°32′E) Jinja^65^, (00°46′N 34°01′E) Nagongera^66^ all in Uganda and the Busia/Bungoma area of Western Kenya^38^. All were collected as larvae with rearing as above and bioassays-screened using standard WHO methods. Samples were genotyped for SNPs using a TaqMan SNP genotyping assay for *Vgsc* L1014S^67^, and novel TaqMan assays for the: (1) *CYP4J10* marker (2L: 25,636,722) primers were (forward 5′-ATCACGGTGCAGATCGT-3′) and (reverse 5′-GCTTCAAGAATCTGAATCGC-3′) and SNP specific probes (G: 5′-6FAM-CATGTCACACGTCCACA-3′ and A: 5′-VIC-CATGTCACACATCCACA-3′); (2) *COEAE1D* marker (2L: 20,288,132) primers were (forward 5′-GAGAGTGCAGGAGCTAAGGC-3′) and (reverse 5′-CTCCACTTTGACAGATCACTCGAT-3′) and SNP specific probes (G: 5′-6FAM-CCTATCTGCATTACCTTT-3′ and A: 5′-VIC-CCTATCTGCACTACCTTT-3′); (3) *CYP4J5* marker (2L: 25635973) primers were (forward 5′-AGCCTGCGCGTGTGATA-3′) and (reverse 5′-CTTCTTCTCCTGTGGTTCGTTTG-3′) and SNP specific probes (G: 5′-6FAM-TTGCCGGAAGGCAGT-3′ and A: 5′-VIC-TTGCCGGAGGGCAGT-3′). Probes carried non-fluorescent quenchers at the 3′ end. Assays were performed in a 10 μl volume containing 1x Sensimix (Bioline), 1x primer/probe mix and 1μl template DNA with a temperature profile of 95 °C for 10 min followed by 40 cycles of 92 °C for 15 s and 60 °C for 1 min on an Agilent MX3005 real-time PCR machine. VIC and FAM fluorescence was captured at the end of each cycle and genotypes called from endpoint fluorescence using Agilent MXPro software. Genotype-phenotype associations were examined using χ^2^ tests and odds ratios.

## Electronic supplementary material

Supplementary Figures

Supplementary Tables
