# Supplementary material for: Candidate-gene based GWAS identifies reproducible DNA markers for metabolic pyrethroid resistance from standing genetic variation in East African Anopheles gambiae
Source: Sci Rep. 2018 Feb 13;8:2920. doi: 10.1038/s41598-018-21265-5 (PMC5811533; doi:10.1038/s41598-018-21265-5)
Supplement: Supplementary file 1 — Supplementary Figures [file 41598_2018_21265_MOESM1_ESM.pdf]

Candidate-gene based GWAS identifies reproducible DNA markers for metabolic pyrethroid resistance from standing genetic variation in East African *Anopheles gambiae*

David Weetman[1†]*, Craig S. Wilding[2†], Daniel E. Neafsey[3], Pie Müller[1,4,5], Eric Ochomo[6,7], Alison T. Isaacs[1], Keith Steen[1], Emily J. Rippon[1], John C. Morgan[1], Henry D. Mawejje[8], Daniel J. Rigden[9], Loyce M. Okedi[10] and Martin J. Donnelly[1,11]

Supplementary Figures

Please see Supplementary information Excel file for Tables S1-S7

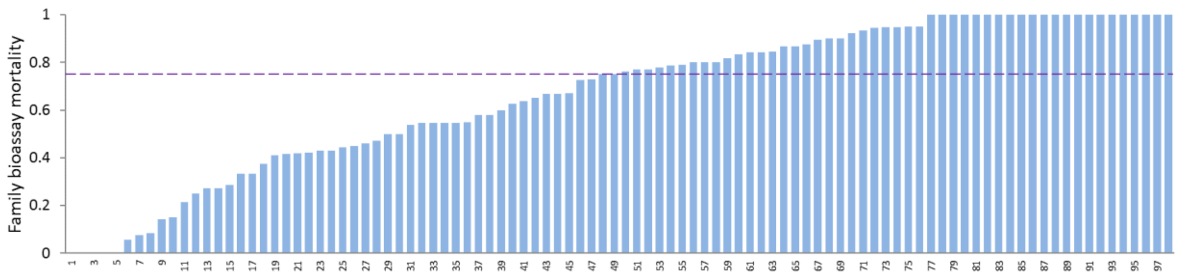

Figure S1. Range of family bioassay mortalities 24 h after a 75 min WHO tube exposure to permethrin. The dashed line shows the median value.

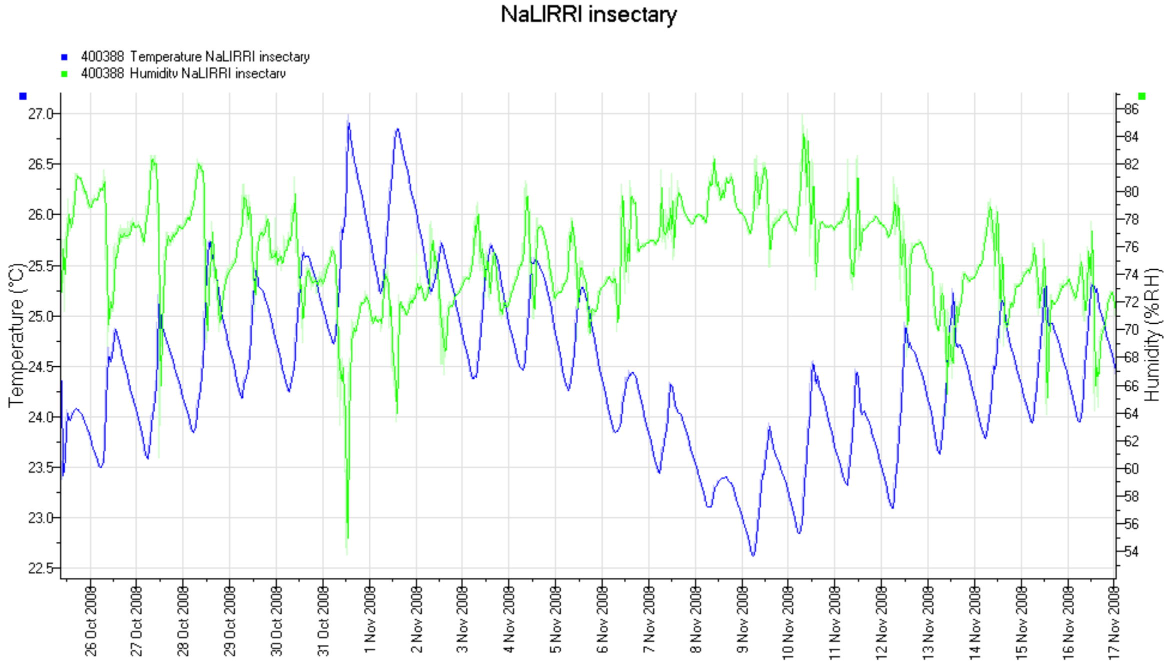

Figure S2. Temperature (blue line) and humidity (green line) variation in the field insectary across the study period (26th October to 17th November)

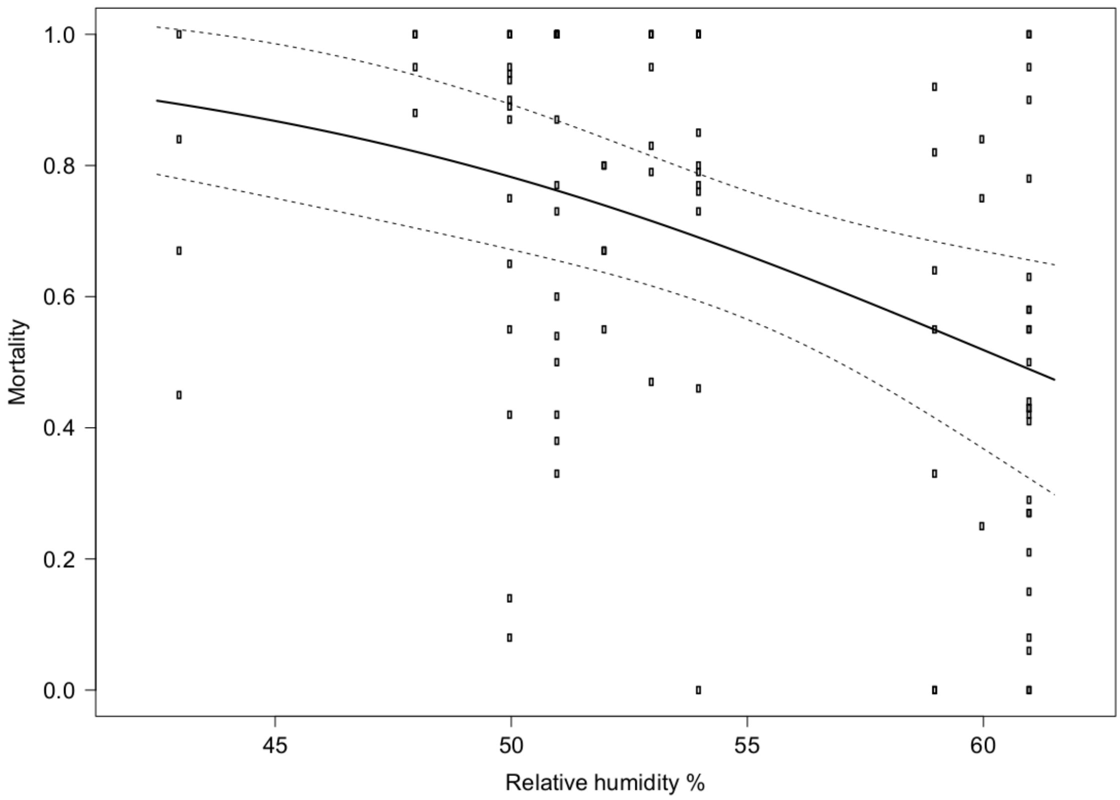

Figure S3. Logistic regression plot (with 95% confidence intervals) illustrating the impact of humidity on family mortality (each point is a family bioassay result).

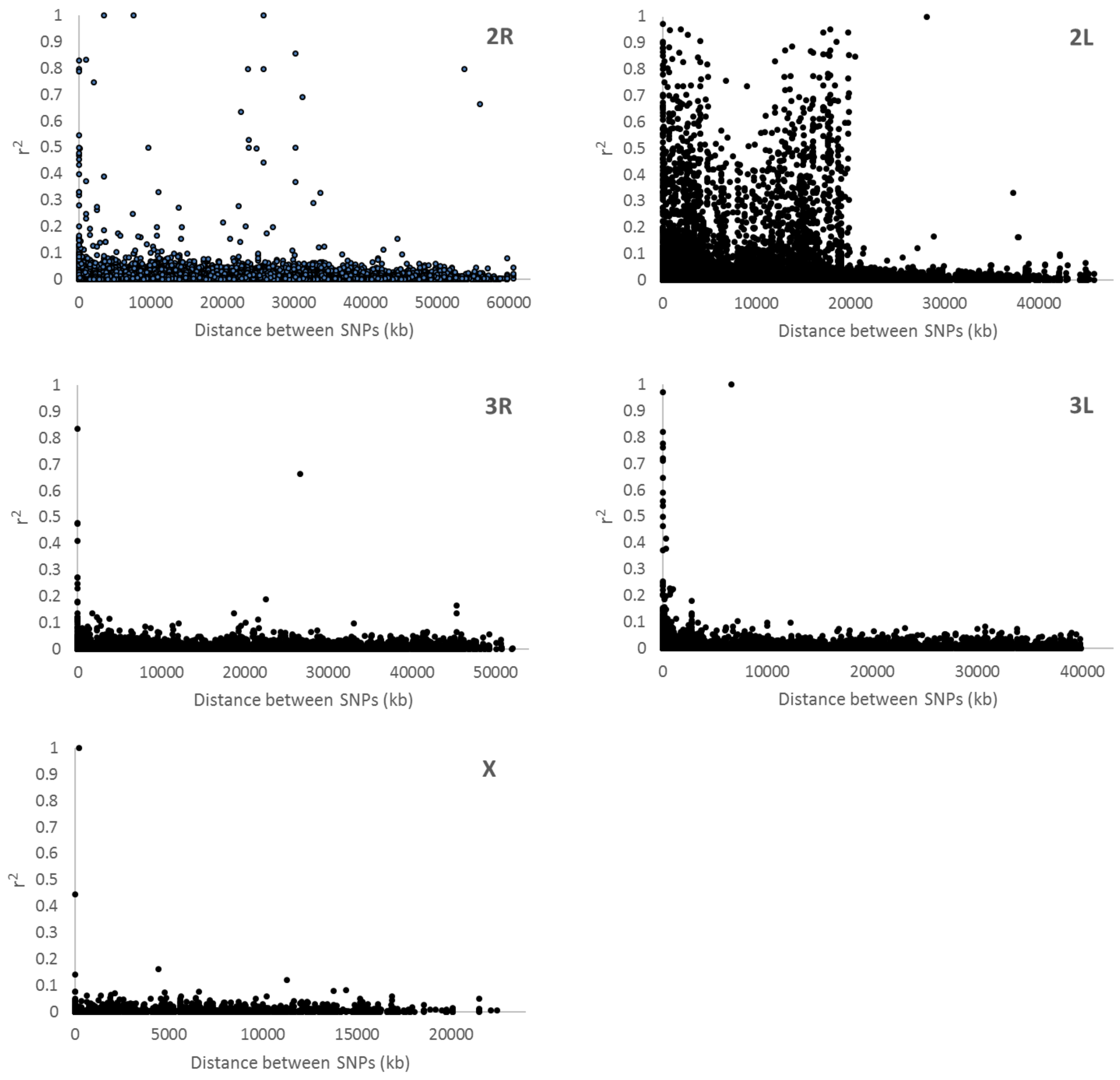

Figure S4. Decay of linkage disequilibrium (measured by r^2) between SNPs at varying pairwise distances on each chromosome.

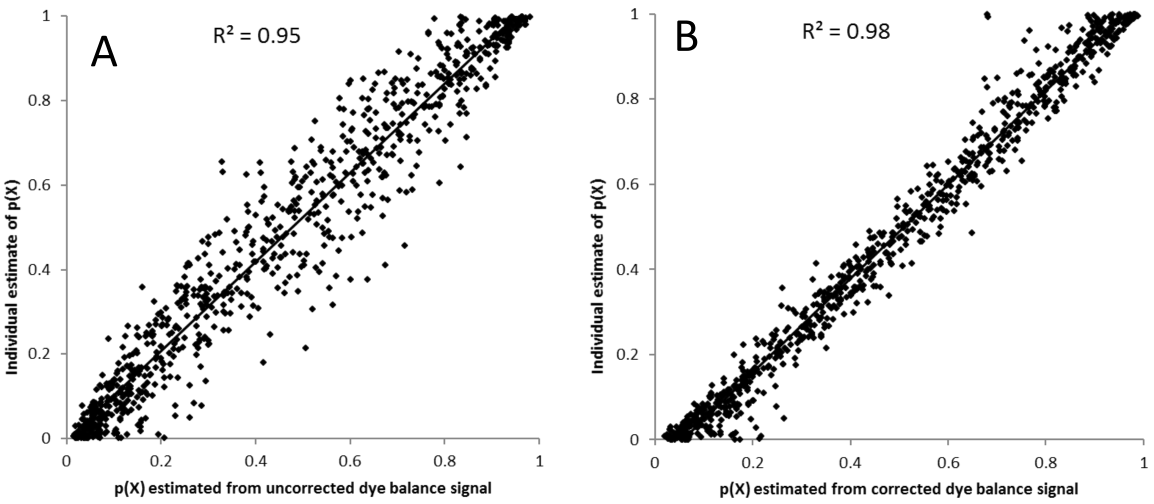

Figure S5. Prediction of population allele frequencies, p(X), obtained from individual genotyping by estimates from pooled genotyping (A) before and (B) after correction for dye bias using the dye-balance signal from heterozygotes

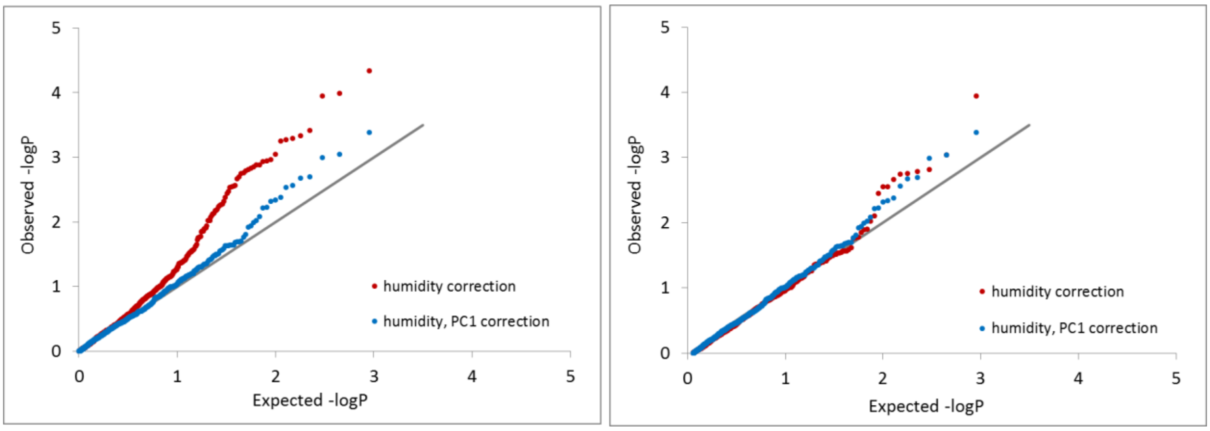

Figure S6. Observed *vs.* expected -log probability plots with and without statistical correction for the prinicipal component reflecting stratification, PC1. (A) all SNPs (B) excluding SNPs from within the 2La inversion region.

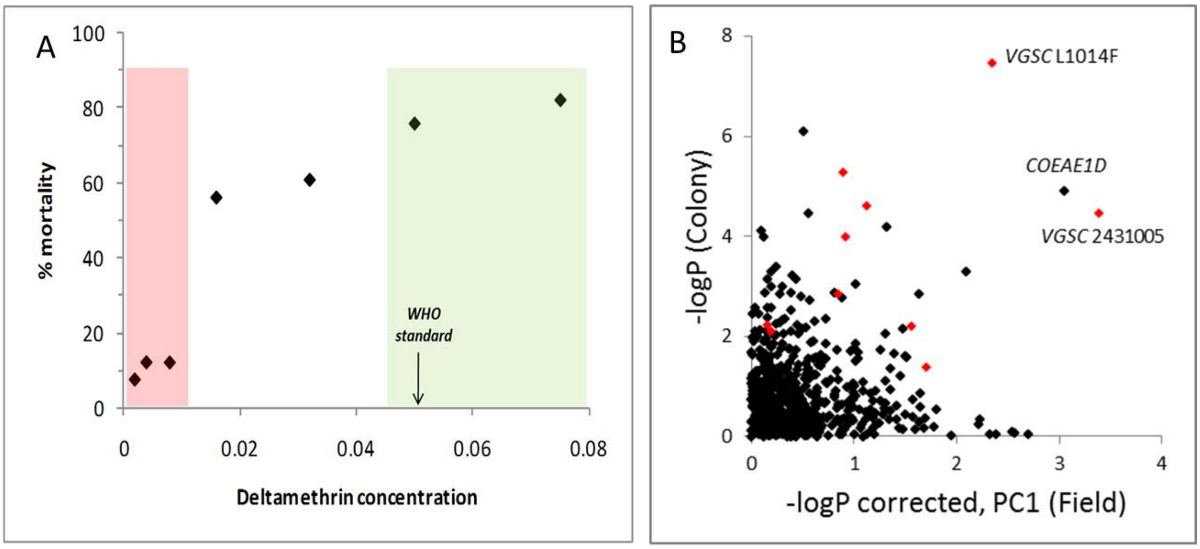

Figure S7. Association analysis in a colony founded from Tororo eggs. (A) Dose-response assay from which resistant (green shading) and susceptible (pink shading) females were chosen for association analysis. (B) Comparison of association test results from permethrin-bioassayed field and deltamethrin-bioassayed colony samples. Red points show SNPs in the *Vgsc* target site gene.

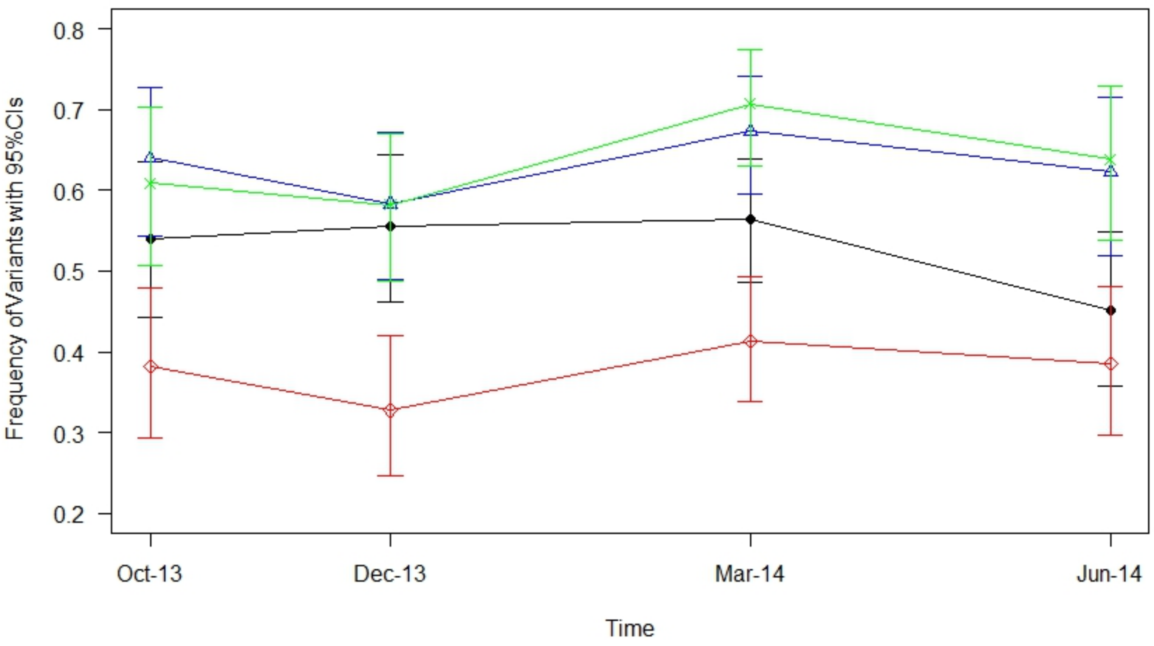

Figure S8. Temporal variation in mean allele frequencies in females (N=237) collected in Nagongera, Tororo in 2013-14, with 95% confidence intervals. Despite apparent covariation in frequencies *CYP4J5* marker genotypes were uncorrelated with 2La variation (Table S5) and *CYP4J10* only marginally. *Coeae1d*, black; *Cyp4j5*, blue; *Cyp4j10*, red; 2La (wild type), green.

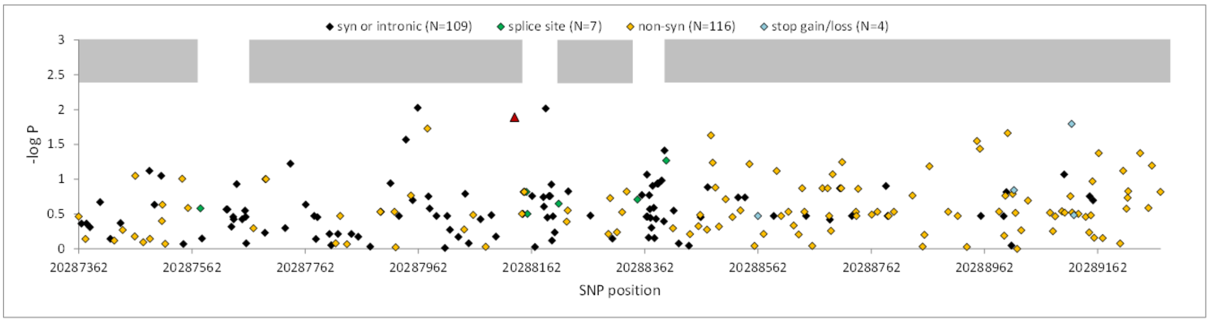

Figure S9. Association analysis of sequence variants in the *Coeae1d* gene. Each point, coloured according to the consequences of a variant at the position (legend), shows a χ^2 association test –logP value for each polymorphic position comparing individuals from resistant and susceptible families (N=24 each), chosen from the extremes of the mortality distribution (see Table S6b for details). Shaded blocks show positions of exons. The associated SNP (20288132) originally identified is shown as a red triangle.
